# Efficacy of an educational multimedia in promoting public health literacy on age-related cognitive-communicative health: a randomized controlled trial

**DOI:** 10.1038/s41598-025-16857-x

**Published:** 2025-09-25

**Authors:** Shreya Shetty, Aysha Rooha, Aarushi Soni, Nidhi Lalu Jacob, Gagan Bajaj, Vinitha Mary George, Jayashree S. Bhat

**Affiliations:** 1 Department of Audiology and Speech Language Pathology, Kasturba Medical College Mangalore, Manipal Academy of Higher Education, Manipal, India; 2 Department of Audiology and Speech-Language Pathology, National Institute of Speech and Hearing, Trivandrum, Kerala India; 3 Department of Audiology and Speech Language Pathology, Nitte Institute of Speech and Hearing, Deralakatte, Mangalore, Karnataka India

**Keywords:** Health literacy, Cognition, Educational multimedia, Health promotion, Young adults, Patient education, Quality of life

## Abstract

Amid the alarming global forecast of age-related cognitive-communicative disorders like dementia, Speech-Language Pathologists (SLPs) are poised to play a crucial role in dementia-related services, ranging from counselling to end-of-life care. A key step in this direction is enhancing public health literacy regarding optimal age-linked cognitive-communicative health. Given the potential of educational multimedia (EM) programs to promote public health literacy, and the recent development of one such EM in our research, this study aimed to evaluate the efficacy of an SLP-designed and facilitated EM in changing young adults (YAs) knowledge and beliefs about cognitive health in the Indian context. In a randomized controlled trial, 220 YAs aged 18–25 years were equally and randomly assigned to either the experimental group (EG) or the active control group (CG). The EG viewed an EM on cognitive health, while the CG watched an animated fictional video. Both groups completed a retrospective pre-post survey evaluating self-perceived knowledge, factual knowledge, and beliefs about cognitive health. The effect of the EM was analyzed using mixed-model ANOVA. A significant improvement in the EG compared to the CG across all three domains was observed. These findings highlight the efficacy of the EM in enhancing understanding and attitudes toward cognitive health among YAs. The EM shows promise as a structured cognitive health education tool, fostering awareness, encouraging preventive measures, and potentially contributing to early detection and intervention of cognitive health issues. This study aligns with the emerging role of SLPs in educating communities about cognitive-communicative health and well-being.

## Introduction

According to the Global Burden of Diseases (GBD) 2019 Dementia Forecasting Collaborators, an estimated 57.4 million people worldwide were affected by dementia in 2019, a number projected to rise to 152.8 million by 2050^1^. In light of this, a survey by the American Speech-Language-Hearing Association (ASHA) reported that dementia-related services constitute the second largest area of clinical practice for Speech-Language Pathologists (SLPs)^2^. A recently published work outlining the role of SLPs in Mild Cognitive Impairment (MCI) and Dementia-related services has underscored a critical area of responsibility as 'Counselling+' activities, in addition to the standard screening, assessment, and management services^3^. In addition to addressing the psychosocial aspects of cognitive disorders and collaborating with healthcare professionals, individuals, and families to enhance services and optimize outcomes, the 'Counseling+' role for SLPs also integrates a critical focus on prevention of age-linked cognitive communicative disorders and the associated wellness, aimed at mitigating both the prevalence and impact of these conditions^3^. Prevention and wellness related activities include dissemination of knowledge regarding measures for healthy cognitive lifestyle, age-associated cognitive declines, and cognitive pathologies, targeting the community at large. Enhancing cognitive health awareness among the general population has also been emphasized by the 'Global Action Plan on public health’s response to Dementia’^4^. As such, it may be a logical and necessary task for SLPs to focus on enhancing public health literacy (HL) about cognitive health among typically aging adults as part of our professional responsibilities.

HL is an important predictor of health outcomes since it defines the capacity of an individual to receive, process, communicate information related to basic health decisions^5^. Individuals with low HL are associated with higher hospitalization rates, developing more medical conditions^6^ and poor adherence to medication^7^. Recent studies indicate a significant association between low HL and high risk of developing Mild Cognitive Impairment (MCI)^8,9^ and Alzheimer’s disease^10^. Poor HL correlates with reduced cognitive abilities across many of its domains^9,11–13^. On the contrary, optimum HL is associated with increased participation in cognitive, social, and physical activities^13,14^. Individuals with high levels of HL promote and maintain good health not only for themselves but their families and communities^15^. Consequently, possessing good cognitive HL is both a personal and social resource, yielding society benefits thereby prompting policy makers and the health care system to provide adequate, appropriate, and accessible information to the society^16^. Therefore, prioritizing the enhancement of cognitive health literacy is paramount for facilitating wellness and preventive measures.

A wide range of studies have been carried out with the aim of advancing knowledge and beliefs pertaining to cognitive health literacy. In this regard, Aihara and Maeda^17^ investigated the effectiveness of the "Dementia Supporter Caravan" campaign in Japan, which included in-person classes educating on dementia. Heger et al.^18^ assessed the impact of an e-health promotion campaign called "My Brain Coach," focusing on three themes: maintaining a healthy diet, regular exercise, and fostering curiosity. Farina et al.^19^ explored the influence of dementia awareness classes on changing attitudes toward dementia, while Askari et al.^20^ aimed to evaluate a dementia awareness campaign that utilized the Community-based Participatory Research (CBPR) model to develop training materials for social workers. The outcomes of these studies collectively affirm the positive impact of HL programs in shaping knowledge and beliefs related to cognitive health.

While prior research has primarily focused on improving cognitive HL among aging populations, there is a relative lack of emphasis on enhancing cognitive health awareness and skills among younger adults. Developing these skills and safeguarding mental well-being should begin at an early life stage to yield greater long-term benefits. Research has demonstrated that cognition enhancing activities undertaken during young adulthood are associated with improved cognitive functioning in later years^21,22^. Promoting cognitive health literacy among this demographic is essential, as they represent the future population at increased risk of cognitive pathologies, making them a crucial target for preventive interventions. Thus, proactive initiatives to enhance cognitive health in young adulthood can help mitigate the risk of cognitive disorders in old age^23^. Educating young adults (YAs) can yield positive impacts not only on their personal health, but also on the well-being of their families and communities, as they can contribute to the cognitive functioning of those around them^24^. Additionally, cultivating an attitude of promoting cognitive well-being during young adulthood can help mitigate cognitive deficits associated with acquired brain injuries, benefiting their current cognitive functioning as well^23^.

Innovative digital media tools hold significant potential to enhance cognitive HL among YAs, given the recent surge in internet accessibility. As of 2022, online usage was higher among individuals aged 15–24 years^25,26^. One of the most widely used digital media for promoting HL in the recent years is an Educational Multimedia (EM). EM-based programs can enhance communication and educational experiences of individuals with limited HL by decreasing reliance on reading skills, and instead providing information through visual aids and audio presentations. EM have been shown to better hold participants’ interest, enhance their experience, satisfaction, knowledge, and behavior change while also improving information retention^27,28^. While generic health promotion programs and campaigns frequently encounter challenges in engaging diverse communities due to the oversight of unique cultural and linguistic aspects within segmented populations^29^, a well-designed multimedia provides flexibility in its delivery and can be tailored to meet linguistic, cultural, and physical needs of diverse populations.

In the Indian context, one such promising digital health tool such as EM has potential for wide reach and impact, given the rapid pace of digitization in the country with over 900 million internet users^30^. Recognizing this potential of EM tools in India, one of our recent projects involved a team of SLPs, with expertise in cognitive-communication, designing a comprehensive, evidence-based EM aimed at promoting cognitive well-being in the Indian population^31^. This resource, carefully tailored to the linguistic and cultural context of its target audience, demonstrates great promise in supporting public health initiatives focused on cognitive health^31^. Following the demonstration of robust content validity, high levels of understandability, and strong actionability, the next step will be to evaluate its real-world impact in enhancing public health literacy about cognitive health among diverse demographic groups. Empirical evaluation of this resource could offer concrete evidence of its effectiveness in advancing cognitive well-being, moving it from theoretical potential to proven utility in practice. This EM could then be advocated among clinical practitioners in the field of cognitive sciences as a structured educational intervention aiming to enhance cognitive health literacy.

## Materials and methods

The research followed a randomized controlled trial (Clinical Trail Registration Number: CTRI/2022/08/044785 registered on 22.08.2022) and was conducted between January 2022 and February 2023. Ethical approval was obtained from the Institutional Ethics Committee (IEC KMC MLR 05-2022/171). All methods and procedures were performed in accordance with the relevant guidelines and regulations.

### Participants

The study recruited healthy YAs aged 18–25 years^32^, who were pursuing non-health-related bachelor’s degrees at an institute in Mangalore, India. Those with a history of neurological, psychological, or psychiatric disorder, and uncorrected hearing or vision were excluded. Functional disability among the participants was assessed using the WHODAS 2.0^33^. A total of 220 YAs met the selection criteria and were recruited for the study. Participants were randomized in 1:1 ratio to either experimental group (EG) (n = 110) or active control group (CG) (n = 110) using the block size of four and the sealed envelope approach. Six respondents from EG and 34 from CG had incomplete questionnaire responses and thus excluded. Thus, the total number of responses from EG was 104 and CG was 76. Individuals in EG viewed the EM related to cognitive health, whereas those in the CG viewed an animated fictional video lacking any educational content about cognitive health. Informed consent and demographic details were obtained at the beginning of the study. Appropriate permissions were obtained from the university authorities for recruitment of the participants.

### Intervention

**Fig. 1:**
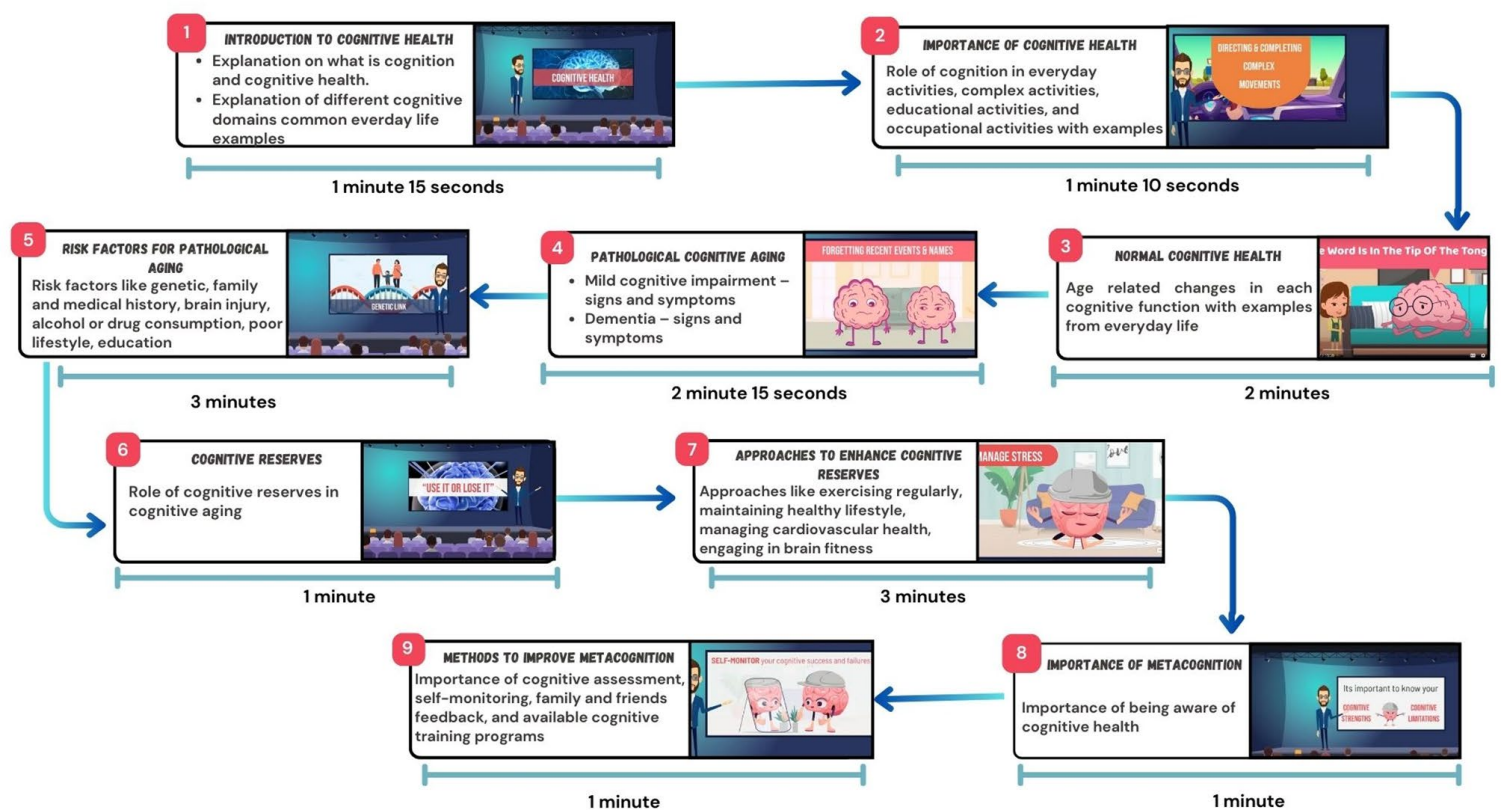
Description of the educational multimedia.

The EM on cognitive health developed in our previous work^31^ was selected as the EM intervention for the EG in the present study due to its comprehensive coverage of cognitive health topics and favorable validation scores it received in both scientific and technical evaluations, as assessed by stakeholders such as SLPs and representatives of general public. The development of this educational content is guided by theoretical foundations, including the integrated HL framework, the cognitive health module provided by the National Institute of Aging, and scientific methodologies such as the modified nominal group technique. The total duration of the EM on cognitive health is 15.38 min revolving around key aspects related to cognitive aging, MCI and dementia, cognitive reserves and metacognition. The components of the EM, including theme-specific details, approaches employed to convey the content, and the duration of each segment, are outlined in Fig. 1. This multimedia disseminates the information in an animated form featuring a central character, Dr. Cognition, who delivers a 'TED-like’ talk titled ‘Towards healthy cognitive aging’ in an engaging and interactive manner. The interaction between Dr. Cognition and the audience serves as a crucial element in keeping viewers engaged. The content of the EM possesses a content validation index of 0.93 and 0.86, and ratings of 92.8% and 98.8% for understandability and 100% for actionability from SLPs and general public.

The CG viewed a pre-existing animated fictional video in which a central character attempts to start small food-related businesses from his home. The video featured simple English dialogues spoken solely by the central character and depicted goal-directed sequences delivered in a neutral tone. It contained no content related to health, cognition, or aging. This video was selected to closely match the experimental video in terms of animation style and duration.

To evaluate the structural and affective comparability of the videos used for the EG and CG, a pilot validation was conducted using a 5-item Likert scale assessing visual engagement, attention-holding capacity, emotional response, enjoyment, and background score. Two independent groups of YAs (n = 10 each), distinct from the main study participants and exposed to either the experimental or control video, rated these dimensions. Statistical comparisons using the Mann–Whitney U test revealed no significant differences across any parameter: visual engagement (U = 45, p = 0.71), attention-holding capacity (U = 28.5, p = 0.09), emotional response (U = 37.5, p = 0.32), enjoyment (U = 31.5, p = 0.15), and background score (U = 31, p = 0.113). These findings suggest that the two videos were structurally and affectively comparable, apart from their thematic content.

### Instrument to assess knowledge and beliefs about cognitive health

A retrospective pre-post questionnaire was developed based on the framework used in the EM^31^, specifically assessing the attributes such as cognitive domains and importance of cognitive health, cognitive changes in typical and pathological aging, cognitive disorders and risk factors associated with it, and preventative measures for cognitive well-being that it claimed to address. The questionnaire was also informed by elements from the Global Brain Health Survey^34^. Retrospective pre-post questionnaire are known to minimize the potential impacts of response-shift bias and improve the accuracy of measure of change^35^. In this study, participants rated their knowledge and beliefs about cognitive health after the intervention, reflecting on their perspectives before and after viewing the EM content through a retrospective pre-post questionnaire. Six SLPs, with over a decade of experience in cognitive sciences, validated the appropriateness of the questionnaire using a 5-point Likert scale where 1—“High inappropriate/Highly incomprehensible/Highly irrelevant”; and 5—“Highly Appropriate/Highly comprehensive/Highly relevant. Modifications were made based on their recommendations to finalize the questionnaire. The modifications included adding a “Not Sure” option for select questions, presenting pre- and post-ratings within a single table for easier comparison, and incorporating descriptive phrases alongside rating scale options for certain items to enhance clarity. The final questionnaire received an acceptable score of > 0.83 by the examiners.

The final questionnaire comprised 15 questions across three domains: Self-perceived Knowledge (SK), Factual Knowledge (FK), and Beliefs (Bf) regarding cognitive health (Tables 1, 2 & 3). SK had three questions, scored on a 4-point scale (maximum score: 12). FK included seven questions, each scored 0.5, 0.75, or 1, with a total possible score of 63. The belief domain consisted of five questions, rated on four or five points, with a potential total score of 68. Additionally, ten YAs outside the study completed the questionnaire twice to assess test–retest reliability, yielding a satisfactory interclass coefficient of 0.87, indicating good reliability.

**Table 1:** Questions from SK domain.

Questions pertaining to SK *(no knowledge, little knowledge, average knowledge, much knowledge)*
1. How much do you know about cognitive health? 2. How much do you know about Mild cognitive impairment? 3. How much do you know about Dementia?
Scoring: 1–4 (no knowledge—much knowledge) Total Domain Score: 12

**Table 2:** Questions from FK domain.

Questions pertaining to FK	
1. Which of the following functions is related to cognitive health?	
a) Sleep	b) Breathing
c) Ability to pay attention	d) Social perception or judgement
e) Understanding and expressing language	f) Reasoning
g) Digestion	h) Decision making
i) Understanding visual and spatial information	j) Memory
k) Learning	l) Directing and completing complex movements
Scoring: 0-Not at all related, 0.5-Somewhat related, 1-Closely related, 0-Not sure. Scoring for options a, b, and g: 1-Not at all related, 0-Somewhat related, 0-Closely related, 0-Not sure Max score: 12	
2. Cognitive health is important for which of the life activities?	
a) Simple daily routines like bathing, dressing, eating etc	b) Skilled activities like cooking, driving, swimming etc
c) Academic activities like learning	d) Carrying out our job (Employment)
e) Making life decisions (Marriage, Job, Finance management etc.)	f) Social interactions
g) Communication	
Scoring: 1-Very Important, 0.5-Somewhat important, 0-Not at all important, 0-Not sure Max Score: 7	
3. How do the following brain functions change with age?	
a) Thinking speed	b) Attention skills
c) Memory for names	d) Memory for information
e) Learning ability	f) Manipulating information in mind
g) Multitasking	h) Vocabulary
i) Reasoning abilities	
Scoring: 0-Increases, 1-Decreases, 0-Remains unchanged, 0-Not sure. Scoring for options h, i: 1-Increases, 0-Decreases, 0-Remains unchanged, 0-Not sure Max Score: 9	
4. Which of these do you think are symptoms of MCI?	
a) Common cold	b) Forgetting the information
c) Misplacing things	d) Cough
e) Difficulty finding words	f) Problem with attention and concentration
g) MCI does not affect activities of daily living	h) Digestive issues
Scoring: 1-Is a symptom, 0-Not a symptom, 0-Not sure. Scoring for options a, d, h: 0-Is a symptom, 1-Not a symptom, 0-Not sure Max Score: 8	
5. Which of these do you think are symptoms of Dementia? (Is a symptom, Not a symptom, Not sure)	
a) Difficulty in recognizing people	b) Putting things in the wrong place
c) Losing weight	d) Feeling lost in new place
e) Slower thinking	f) Losing track of time
g) Losing their temper easily	h) Hair loss
i) Feeling depressed	j) Feeling extremely tired
Scoring: 1-Is a symptom, 0-Not a symptom, 0-Not sure. Scoring for options c, h: 0-Is a symptom, 1-Not a symptom, 0-Not sure Max Score: 10	
6. How much do the following factors affect cognitive health?	
a) Physical health	b) Diet
c) Physical environment	d) Social environment
e) Education	f) Profession
g) Family income	h) Family and genetics medical history
i) Substances uses	j) Sleeping habits
k) Having objectives that give life meaningful	
Scoring: 1-Strong influence, 0.75-Moderate influence, 0.5-Weak influence, 0-No influence, Not sure Max Score: 11	
7. What stages in life is it important to look after one’s cognitive health?	
a) In the womb (before birth)	b) Childhood (from birth to 12 years)
c) Adolescence (13–18 years)	d) Young adulthood (19–45)
e) Middle age (45–65)	f) Old age (over 65 years
Scoring: 1-Very Important, 0.5-Somewhat important, 0-Not at all important, 0-Not sure. Scoring for option a: 0-Very Important, 0-Somewhat important, 1-Not at all important, 0-Not sure	
Max score: 6 Total Domain score: 63

**Table 3:** Questions from the beliefs domain.

Questions pertaining to Bf	
1. How often do you worry about your cognitive health?	
Scoring: 4–1 (Frequently, Occasionally, Rarely, Never) Max Score: 4	
2. How often do you worry about the cognitive health of your family or friends?	
Scoring: 4–1 (Frequently, Occasionally, Rarely, Never) Max Score: 4	
3. Would you wish to take a simple cognitive test to learn about your present cognitive status and future risks associated with it?	
Scoring: 4–1 (Definitely, Probably, Probably Not, Definitely Not) Max Score: 4	
4. How likely would you do any of the following specifically for your cognitive well-being?	
a) Eat healthier	b) Avoid alcohol
c) Exercise more	d) Avoid smoking
e) Improve sleeping habits	f) Socialize more
g) Do more relaxing activities	h) Play brain games like sudoku, crossword, puzzles
i) Stimulate my brain more (e.g., learn a new language)	j) Sign up for a cognitive fitness app
Scoring: 5–0 (Very likely, Somewhat likely, Already doing, Somewhat unlikely, Very unlikely, Never thought about it) Max Score: 50	
5. Just like fitness training, how likely would you enroll for a cognitive training program to remain cognitively healthy?	
Scoring: 6–1 (Very likely, Somewhat likely, Already enrolled, Somewhat unlikely, Very unlikely, Never thought about it)	
Max score: 6 Total Domain score: 68	

### Procedure

Data collection took place in university auditorium halls, with participants from the EG and CG seated separately in different auditoriums. Investigators provided both groups with a brief introduction to the session’s purpose and schedule, ensuring a distraction-free environment. The EG watched an educational video on cognitive health, while the CG watched a pre-existing animated video of equal duration which was not related to brain health. Both videos were shown on a large screen with audio through loudspeakers. Participants from both groups completed a retrospective pre-post survey on cognitive health immediately after watching the videos. The survey was administered using a paper-and-pen format. To ensure an accurate baseline for assessing participants’ initial knowledge, and to minimize recall bias and overestimation of change, participants were instructed prior to completing the form as follows.

*“For each item on the questionnaire, please reflect on how you would have rated yourself before watching this video on healthy cognitive aging. Then, indicate your level of knowledge after watching the video for the same item. If you’re unsure, you may select the ‘Unsure’ option. There are no right or wrong answers—please respond as honestly as possible.”*

### Data analysis

Data from the retrospective pre-post questionnaire was analyzed using Jamovi software (v2.3.21). Descriptive statistics were used to calculate the mean and standard deviation of the pre- and post-test scores on the SK, FK, and Bf domains. A mixed model ANOVA with a post hoc Bonferroni pairwise analysis was conducted to examine the effects of an EM intervention on knowledge and beliefs related to cognitive health between the EG and CG.

## Results

**Fig. 2:**
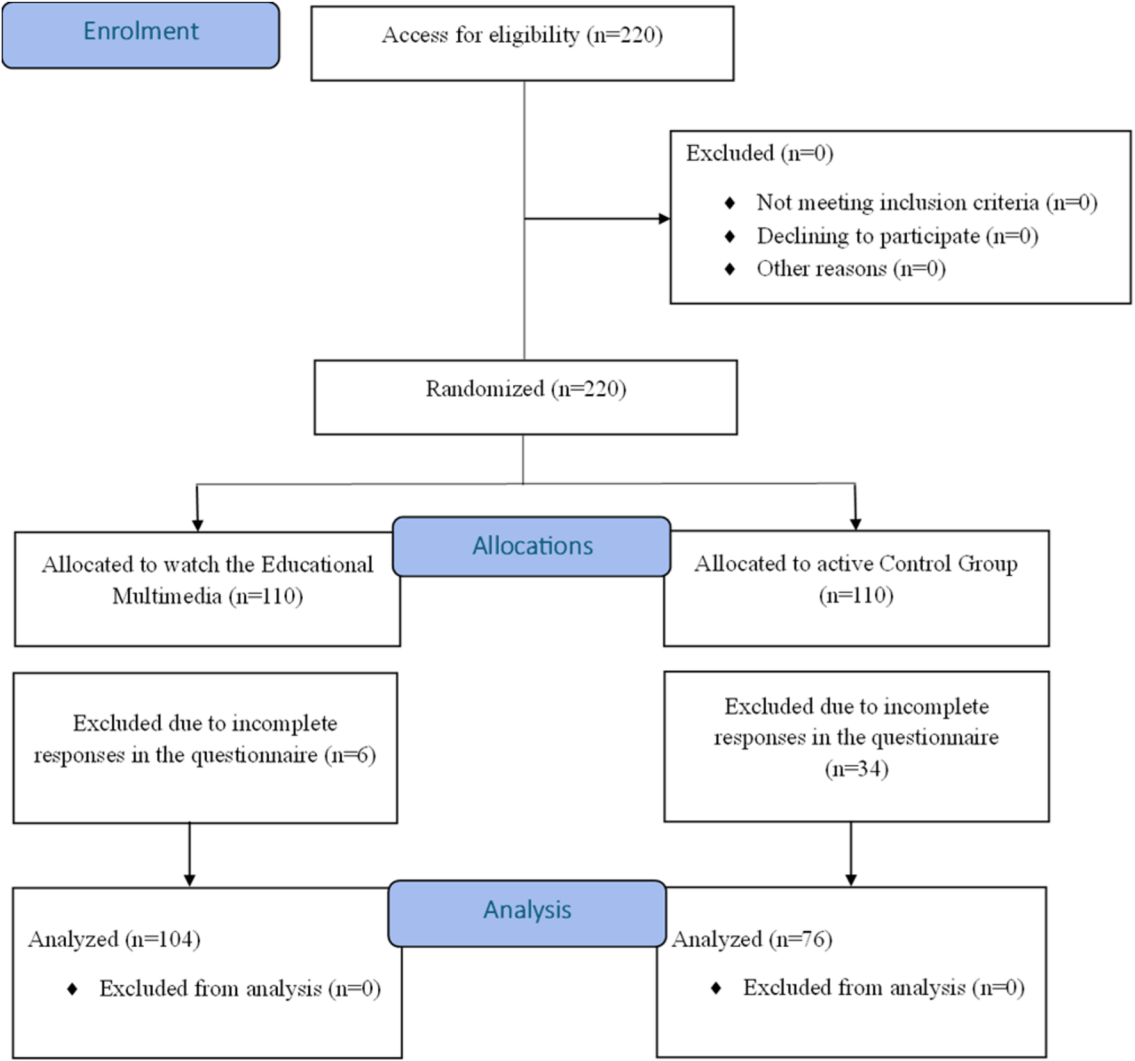
Flow of participants through the evaluation.

The flow of participants through this study is presented in Fig. 2. Participants from both the groups were significantly similar in terms of age [t(178) = − 0.583, p = 0.560], gender [${\chi }^{2}$(180) = 0.989, p = 1], family history of Dementia [${\chi }^{2}$(180) = 0.00251, p = 0.960], acquaintance with Individuals with dementia [${\chi }^{2}$(180) = 0.0588, p = 0.808], prior exposure to multimedia on cognitive health [${\chi }^{2}$(180) = 0.0331, p = 0.856], and acquaintance with health professionals [${\chi }^{2}$(180) = 0.132, p = 0.716]. The demographic details are illustrated in Table 4.

**Table 4:** Demographic details of participants of both the groups.

	Experimental group (n = 104)	Control group (n = 76)
Age	19.63 ± 1.31 years	19.52 ± 1.11 years
Gender	Male: 56 Female:48	Male:41 Female:35
Family history of Dementia/Alzheimer’s	8	6
Acquaintance with individuals with dementia	15	10
Prior exposure to multimedia on cognitive health	9	6
Acquaintance with health professionals	20	13

### Effect of the educational multimedia on knowledge and belief about cognitive health

**Fig. 3:**
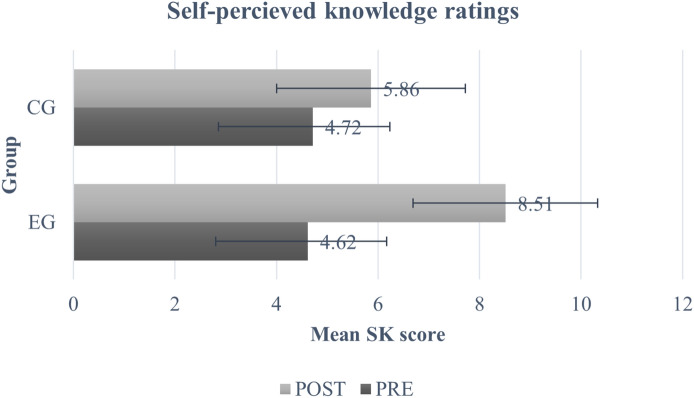
Mean and SD of self-perceived knowledge ratings. Error bars represent standard deviation.

**Fig. 4:**
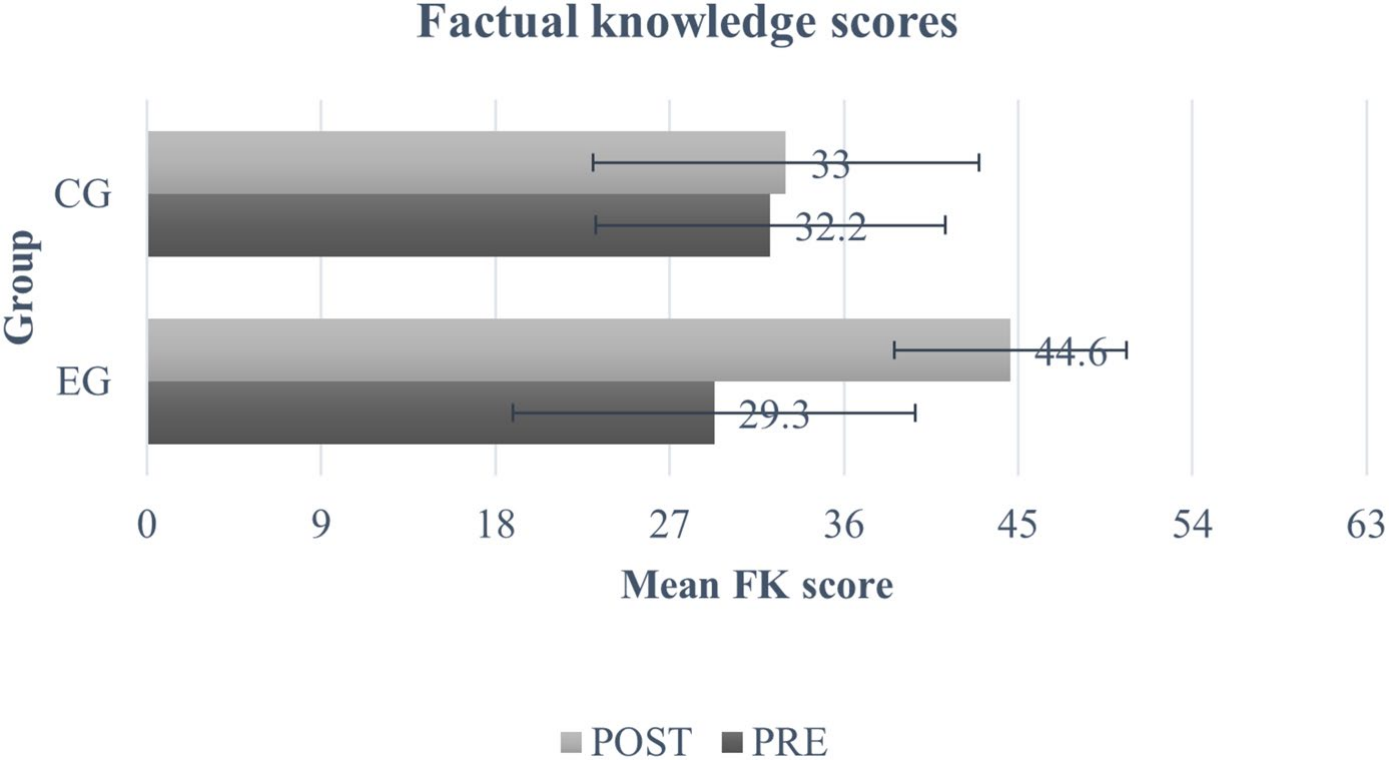
Mean and SD of factual knowledge scores. Error bars represent standard deviation.

**Fig. 5:**
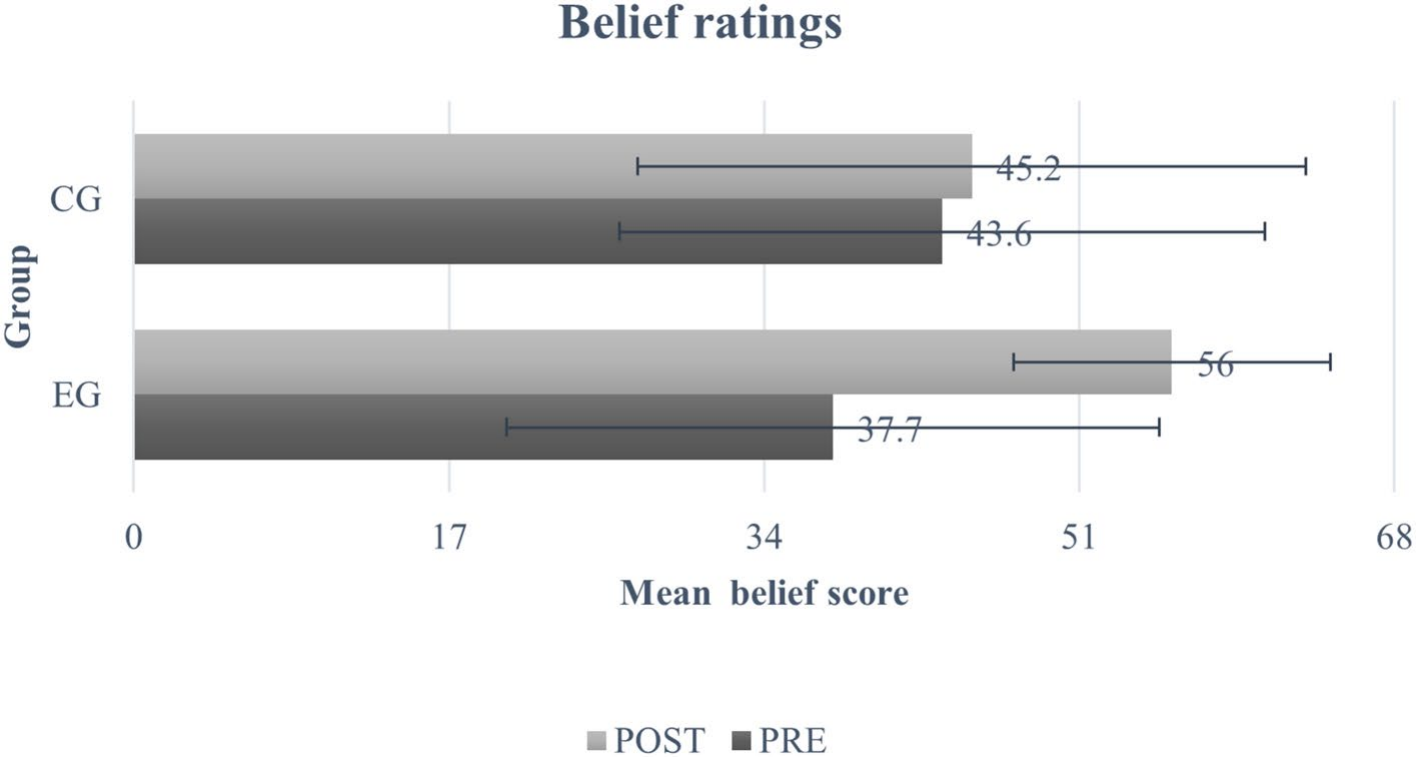
Mean and SD of the belief ratings. Error bars represent standard deviation.

The mean scores for both groups across both time points on all the three domains are provided in Figs. 3, 4 and 5. There were no statistically significant differences in baseline scores between both groups across any of the three domains (SK (t = − 0.47, p = 1.0), FK (t = − 1.92, p = 0.34), and Bf (t = − 2.23, p = 0.16)) (Table 5). The results obtained from the mixed model ANOVA revealed a significant improvement at post-test for participants in the EG as compared to CG for SK domain (F(1,178) = 106, *p* < 0.001, *η*^2^ = 0.89) (Fig. 3 and Table 5), on measures of FK (F(1,178) = 130, *p* < 0.001, *η*^2^ = 0.113) (Fig. 4 and Table 5), and in the Bf domain (F(1,178) = 57.2, *p* < 0.001, *η*^2^ = 0.060)) (Fig. 5 and Table 5). Further, the post-hoc tests revealed significant improvement in the scores of the three domains for EG (SK (t = − 22.34, p < 0.001), FK (t = − 18.57, p = 0.001), and Bf (t = − 12.75, p < 0.001)) and SK domain for CG (t = − 5.55, p < 0.001). There was no significant change in FK scores (t = − 0.88, p = 1.0) and Bf ratings (t = − 0.96, p = 1.0) for the CG.

**Table 5:** Mixed model ANOVA and p-values of pairwise analysis of pre-training and post-training on efficacy of educational video in experimental group and control group.

Domains	*F* (1, 178)	*P*	η^2^	E_pre_ vs C_pre_		E_pre_ vs E_post_		C_pre_ vs C_post_	
				T	*p*	T	*p*	T	*p*
SK	106	**< 0.001**	0.089	− 0.468	1.0	− 22.34	**< 0.001**	− 5.549	**< 0.001**
FK	130	**< 0.001**	0.113	− 1.916	0.341	− 18.572	**< 0.001**	− 0.882	1.0
Bf	57.2	**< 0.001**	0.060	− 2.233	0.161	− 12.758	**< 0.001**	− 0.957	1.0

Highlight represents p < 0.05.

## Discussion

The results of the present study indicate a significant increase in SK, FK, and Bf scores for the EG following the intervention. These findings align with earlier studies showing the effectiveness of educational interventions on cognitive health, including cognitive aging, dementia, and Alzheimer’s, among adolescents, middle-aged adults, and the elderly^17–20^.

The EG’s improvement in the SK domain suggests that participants perceived the educational intervention as enhancing their knowledge on cognitive health, mild cognitive impairment, and dementia. This is significant considering the general lack of understanding about dementia and its prevention among the public^36–38^. The key factors driving user acceptance of any health literacy videos are the informational value, relevance, and social connection it offers^39^. The EM used in this study is reported to be strategically designed to include dyadic interactions between characters, and relatable real-life examples depicting the importance of cognitive health and the signs of cognitive disorders^31^. In the present study, such strategic components likely influenced participants’ acceptance of the EM, contributing to the observed increase in SK scores. The increase in SK scores may indicate that participants perceived an improvement in their ability to comprehend and use information related to cognitive health following the EM, suggesting enhanced self-efficacy. Self-efficacy, or one’s belief in their capacity to action^40^, is a key factor influencing health behaviors^41^. Individuals with higher self-efficacy tend to be more likely to adhere to recommended self-care practices^42^. Thus, this enhanced SK score may aid in promoting cognitive well-being practices among the YAs. However, follow-up assessments to capture their practice patterns post-intervention may be necessary to confirm this. Future studies could focus on incorporating these follow-up assessments to better understand the long-term impact of the EM.

The increase in FK scores indicate enhanced understanding of cognitive functions, their significance in daily life, age-related cognitive changes, signs of impairment, risk factors for cognitive decline, and the importance of cognitive health across the lifespan. The increase in FK ratings could be attributed to the rich and holistic content about cognitive health included in the EM. The EM had been developed with an intention to promote cognitive health and prevent cognitive disorders. It emphasized key themes centered around significance of preserving cognitive function, assessing risk factors that could jeopardize cognitive health, evaluating one’s cognitive status in relation to established norms, and being cognizant of appropriate preventive measures to mitigate the risk of developing cognitive disorders in the future. The EM content has been reported to have high content validity as deemed by experts and general public representation^31^. The results of the present study further reinforce these reports. Moreover, creating a mental representation through multisensory experiences increases the likelihood that the information will be remembered and therefore, followed^43^. Thus, the multimedia format of the intervention that aided in participants comprehension and retention of the content could have also contributed to these results. To confirm this, further studies may be needed to compare the effects of EM with non-EM formats. The FK ratings are a testimony to the appropriateness and usefulness of the EM intervention in enhancing people’s knowledge about cognitive health. This enhancement in their FK may favorably influence their ‘need for cognition’, the propensity to engage in cognitively stimulating pursuits^44^. It could also facilitate the timely detection of cognitive impairments such as MCI and Dementia in themselves as well as in the older adults in their social circles. Furthermore, it may empower them to proactively address risk factors associated with cognitive health and make well-informed lifestyle decisions that could positively impact their cognitive functioning over the long term. Overall, this enhanced knowledge equips YAs with resources and motivation to maintain and enhance their cognitive well-being throughout their lifespan.

Bf ratings suggest that the intervention effectively motivated participants to seek cognitive assessments, adopt healthy cognitive lifestyles, and engage in cognitive training programs. The Health Belief Model^45^ suggests that one’s likelihood of engaging in healthy behaviors is based on their perceived susceptibility and severity of disease, as well as their perceived benefits and barriers to preventive actions. For example, people are more inclined to adopt cognitively healthy habits if they feel at risk of cognitive decline (perceived susceptibility), recognize the importance of cognitive skills for daily functioning, and understand the serious implications of cognitive disorders on overall health (perceived severity). Additionally, their cognitive practices are influenced by their beliefs about the effectiveness of preventive measures (perceived benefits). The Health Belief Model^45^ and HL Skills Framework^36^ indicate that health literacy can shape behavioral outcomes by influencing people’s motivations and beliefs about health. Notably, the educational materials used in the present study directly addressed the significance of cognitive health in daily life, relevant risk factors, symptoms of cognitive disorders, and various preventive strategies. Consequently, the increase in participants’ Bf ratings may be attributed to their enhanced cognitive health literacy, as reflected in their SK and FK scores.

The present study specifically was addressed for YA population as a positive influence on them could have optimistic effects on the society in the coming years^26^. Majority of the behaviors one practices are established in the young adulthood^46^ making it essential to educate YAs about the benefits of HL and lack thereof, as they are at a crucial stage of their lives where the behaviours established today will affect their decisions later in adulthood. Moreover, YAs are increasingly taking on more responsibility for making health-related decisions and acting as “primary caregivers” for themselves and their families^47^. Although theoretical knowledge alone may not inspire lifestyle changes or promote health, it may contribute in better understanding of how one’s choices impact their well-being^48^. Moreover, research has linked greater knowledge with greater interest and motivation to decrease the risk of cognitive disorders^18,49,50^. In this essence, the present attempt could be a small step towards fostering greater interest, motivation, and knowledge about cognitive health and well-being, which could potentially offer some relief to the alarming statistical predictions of cognitive disorders^51,52^. The rising demand for SLP services in cognitive-communicative health, coupled with the growing recognition of the crucial role SLPs can play in prevention and wellness efforts^3^, our present work highlights the value and utility of the EM resource that has been developed. This comprehensive and scientifically-grounded EM could serve as a valuable guiding tool and resource for SLPs as we work to promote cognitive health and well-being within our clinical practice and community-based initiatives.

## Limitations and future directions

The present research primarily focused on immediate changes in knowledge and beliefs related to cognitive health following the EM and lacks assessment of changes in participant’s practice patterns concerning cognitive health. However, it is acknowledged that participants intentions may serve as influential factors in shaping their behavior^53^, offering a potential indication of their practice patterns. Future studies could directly explore its effect on enhancing self-efficacy and encouraging adoption of healthy cognitive practices by conducting a follow-up assessment through booster sessions and follow-up surveys. Further, as the study focused on core symptoms of dementia, signs such as hair loss and weight loss were not considered as symptoms during analysis, despite certain evidence suggesting their association with dementia. The assessment of factual knowledge regarding cognitive changes with aging broadly encompassed reasoning and memory. However, evidence on changes in these abilities is heterogeneous, with different types of reasoning and memory domains showing distinct trajectories. Future research should aim to assess domain-specific knowledge, such as memory for names, memory for faces, verbal reasoning, social reasoning, analogical reasoning, familiar reasoning, etc. With regard to the data collection method, the present study used a retrospective pre-post questionnaire, which may present certain challenges such as recall bias, overestimation of change, and lack of a clear baseline to gauge participants’ initial knowledge and attitude. Although these limitations were addressed by providing appropriate instructions to participants, future studies could consider incorporating this questionnaire at two different time points—before and after the educational multimedia. Also, designing custom, closely matched placebo videos for control groups may be an important methodological consideration for upcoming research. Further, as prior experience with any form of multimedia education may influence engagement and responsiveness to the intervention, screening for it could be considered in future iterations of the study. Research evaluating the efficacy of the EM across a broader range of demographics such as age, clinical conditions, and educational and linguistic backgrounds is warranted, particularly through the use of culturally and linguistically appropriate material.

## Conclusions

The present research aimed to evaluate the efficacy of an EM intervention on YAs self-perceived knowledge, factual knowledge, and beliefs about cognitive health using a retrospective pre-post questionnaire. The results confirmed a significant improvement in all three domains among the EG compared to the CG, suggesting that the selected EM effectively enhanced YAs understanding and attitudes towards cognitive health. The EM used in the present study could serve to spread awareness about cognitive health, encourage the general population to take preventive measures, and promote early detection and intervention of cognitive health issues. Unlike traditional public health initiatives, EM can reach large and remote audiences with minimal recurring costs, making it a cost-effective tool for health promotion. Its flexibility and low training requirements also allow for seamless integration into routine health education, community outreach programs, and clinic waiting areas. The present research aligns with the growing responsibilities of SLPs in cognitive well-being, offering a tool that can be integrated into our ‘Counselling+’ services, a crucial aspect of our evolving professional role. Future studies could examine changes in participants’ behaviour and explore how multimedia can promote healthy cognitive practices while also considering more diverse demographics and linguistically suitable approaches.

## Supplementary Information

Supplementary Information.

## Data Availability

The data that supports the findings of this study are available from the authors upon reasonable request.
