# Supplementary material for: Efficacy of an educational multimedia in promoting public health literacy on age-related cognitive-communicative health: a randomized controlled trial
Source: Sci Rep. 2025 Sep 25;15:32915. doi: 10.1038/s41598-025-16857-x (PMC12464230; doi:10.1038/s41598-025-16857-x)
Supplement: Supplementary file 1 — Supplementary Information. [file 41598_2025_16857_MOESM1_ESM.docx]

**RESEARCH PROTOCOL**

**Project Summary**

Dementia rates are expected to nearly triple by 2050, highlighting a global health challenge and the urgent need for preventative strategies^1^. Enhancing public health literacy on cognitive wellness is crucial to reduce the impact of cognitive decline and support healthier aging^2^. Aligning with the SLPs’ growing role in advancing public health education on cognitive wellness^3^, this study aims to empirically evaluate the effectiveness of an SLP-designed educational multimedia program developed within our research to improve cognitive health knowledge and beliefs among young adults in India. Specifically, it assesses the program’s impact on young adults' knowledge and belief of cognitive health. Conducted as a single-blind, randomized controlled trial from January 2022 to February 2023, the study recruited 220 healthy adults aged 18 to 25 years, randomized 1:1 into an experimental group (EG) (n = 110) and an active control group (CG) (n = 110). The EG viewed an educational video on cognitive health, while the CG watched a similar-length animated video unrelated to brain health. The participants rated their cognitive health knowledge and beliefs post-intervention via a retrospective pre-post questionnaire covering Self-perceived Knowledge (SK), Factual Knowledge (FK), and Beliefs (Bf). The impact of the educational multimedia program was analyzed using a mixed-model analysis of variance to examine the immediate responses of participants after viewing the content. The study aims to evaluate the impact of the educational multimedia program on young adults' self-perceived knowledge, factual knowledge, and beliefs about cognitive health. The results are expected to demonstrate improvements across these three domains, highlighting the potential of such educational resources to enhance cognitive health literacy among young populations.

**General Information**

- **Protocol title:** Efficacy of an Educational Multimedia in Promoting Public Health Literacy on Age-Related Cognitive-Communicative Health: A Randomized Controlled Trial
- **Name and address of the funder:** Department of Science and Technology (DST) under Cognitive Science Research Initiative (CSRI), Government of India (DST/CSRI/2018/29).
- **Name and title of the investigators who are responsible for conducting the research, and the address and telephone numbers of the research site, including responsibilities of each:**

*Investigators:* Shreya Shetty (Masters Student), Aysha Rooha (Research Scholar), Aarushi Soni (Research Scholar), Nidhi Lalu Jacob (Research Scholar), Gagan Bajaj (Associate Professor), Vinitha Mary George (Professor (I/C) & HOD), Jayashree S Bhat (Professor)

*Address of Research site:* Cognitive Communicative Lab, Department of Audiology & Speech Language Pathology, Kasturba Medical College, Mangalore, Manipal Academy of Higher Education, Manipal, Karnataka, India

*Responsibilities of each:* All authors (AR, SS, AS, NLJ, GB, VMG, JSB) were involved in Conceptualization and designing the Methodology of the research idea. AR, SS and NLJ involved in the Investigation. Formal analysis by AR, SS, AS, GB. Project administration and Resources provided by GB, VMG and JSB. AR, SS, AS, and GB wrote the original draft. All authors (AR, SS, AS, NLJ, GB, VMG, JSB) contributed to refinement of the study protocol and approved the final manuscript.

- **Name and address of the clinical laboratory and other medical and/or technical department and/or institutions involved in the research:** Cognitive Communicative Lab, Department of Audiology & Speech Language Pathology, Kasturba Medical College, Mangalore, Manipal Academy of Higher Education, Manipal, Karnataka, India

**Rationale & background information**

The impending and substantial rise in dementia prevalence, projected to nearly triple by 2050, has rendered addressing cognitive health a pressing public health priority^1^. Strengthening population-level health literacy (HL) pertaining to cognitive wellness is essential to empower individuals in making informed decisions regarding lifestyle modifications, age-related cognitive decline, and the risk of cognitive disorders^4^. The "Global Action Plan on Dementia" emphasizes the importance of heightening public awareness, as health literacy significantly influences individuals' capacity to access, comprehend, and act upon health-related information^2^.

Young adulthood is a critical period for establishing lifelong behaviours, making it an ideal time to introduce HL initiatives. The habits and decisions formed at this stage can have lasting effects on individuals’ health and well-being, both for themselves and, as they grow into adulthood, for those around them. Young adults today are taking on more responsibility in making health-related decisions, often acting as primary caregivers in their families^5–7^. However, limited awareness about cognitive health and preventive practices poses a significant barrier to sustaining cognitive wellness. An educational multimedia (EM) resource tailored to young adults could bridge this gap, equipping them with essential knowledge about cognitive health in an engaging, accessible format. By fostering HL early, such an intervention has the potential to influence health behaviours positively across communities in the years to come.

EM has emerged as an effective tool to improve HL, especially for those with limited literacy skills, by offering visually engaging and accessible information. Studies show EM programs enhance engagement, knowledge retention, satisfaction, and behaviour change, making them particularly relevant for promoting cognitive health^8,9^. In the rapidly digitizing Indian context, with over 900 million internet users^10^, EM holds immense potential to increase outreach. However, few studies have examined the effectiveness of EM-based cognitive health interventions in India, where surveys reveal limited awareness and practices regarding dementia prevention.

As dementia rates are anticipated to surge in India, the need for effective, culturally relevant tools to enhance cognitive health awareness has become critical. EM out as a compelling solution, offering an engaging, accessible means to improve health literacy and empower individuals with essential knowledge for cognitive wellness. Our team has developed and validated an EM resource uniquely tailored to India's cultural and linguistic diversity, addressing crucial health literacy gaps. This study aims to rigorously evaluate the EM's impact on cognitive health knowledge and beliefs, positioning it as a vital tool for fostering proactive, informed approaches to cognitive health.

**Study goals and objectives**

The objective of the present study was to examine the efficacy of the educational multimedia on knowledge and beliefs of young-aged adults about cognitive health.

**Study design**

***Type of study:*** Randomized Controlled Trial

***Research population or the sampling frame:*** The study recruited healthy young adults aged 18 to 25 years^11^, who were pursuing non-health-related bachelor's degrees at an institute in Mangalore, India. Those with a history of neurological, psychological, or psychiatric disorder, and uncorrected hearing or vision were excluded. Functional disability among the participants was assessed using the WHODAS 2.0^12^. A minimum of 174 YAAs was recommended for the study based on the following sample size formula (n=2(𝑍𝛼+𝑍𝛽)^2^ϭ^2^)/d^2^) with respect to the study done by Lincoln et al.^13^ where, Zα = 1.96 at 95% confidence level, Zβ = 1.28 at 90% power, σ = 3.32, d = 1.12. A total of 220 young adults met the selection criteria and were recruited for the study.

***Expected duration of the study:*** January 2022 and February 2023

**Methodology**

***Randomization and Blinding***

Participants were randomized in 1:1 ratio to either experimental group (EG) (n = 110) or active control group (CG) (n=110) by following a block randomization with a block size of four. Participants were randomly allocated using a sealed envelope approach, with the random allocation sequence. The present study utilized a single-blind design, where the participants were unaware of the group to which they were allocated.

***Intervention***

The EM on cognitive health^14^ was selected as the EM intervention for the present study due to its comprehensive coverage of cognitive health topics and favourable validation scores it received in both scientific and technical evaluations, as assessed by stakeholders such as healthcare professionals and representatives of general public. The development of this educational content is guided by theoretical foundations, including the integrated health literacy framework, the cognitive health module provided by the National Institute of Aging, and scientific methodologies such as the modified nominal group technique. The total duration of the EM on cognitive health is 15.38 mins revolving around key aspects related to cognitive aging, MCI and dementia, cognitive reserves and metacognition. The themes of the EM include – introduction to cognitive health, importance of cognitive health, cognitive health in typical aging, pathological cognitive aging, risk factors for cognitive disorders, cognitive reserves, approaches to enhance cognitive reserves, importance of metacognition, and methods to improve metacognition. This multimedia disseminates the information in an animated form featuring a central character, Dr. Cognition, who delivers a 'TED-like' talk titled ‘Towards healthy cognitive aging’ in an engaging and interactive manner. The interaction between Dr. Cognition and the audience serves as a crucial element in keeping viewers engaged. The content of the EM possesses a content validation index of 0.93 and 0.86, and ratings of 92.8% and 98.8% for understandability and 100% for actionability from SLPs and general public.

***Measurements***

A retrospective pre-post questionnaire was created by modifying items from the Global Health Survey^15^ and Knowledge, Attitude and Practice study on general practitioners in MCI detection and management^16^. Retrospective pre-post questionnaire are known to minimize the potential impacts of response-shift bias and improve the accuracy of measure of change^17^. In this study, participants rated their knowledge and beliefs about cognitive health after the intervention, reflecting on their perspectives before and after viewing the educational multimedia content through a retrospective pre-post questionnaire. This questionnaire was designed based on the framework used in the EM^14^, specifically assessing the attributes such as cognitive domains and importance of cognitive health, cognitive changes in typical and pathological aging, cognitive disorders and risk factors associated with it, and preventative measures for cognitive wellbeing that it claimed to address. Six SLPs, with over a decade of experience in cognitive sciences, validated the appropriateness of the questionnaire using a 5-point Likert scale where 1- “High inappropriate/Highly incomprehensible/Highly irrelevant”; and 5- “Highly Appropriate/Highly comprehensive/High relevant. Modifications were made based on their recommendations to finalize the questionnaire. The evaluation received an acceptable score of >0.83 upon completion. The final questionnaire comprised 15 questions across three domains: Self-perceived Knowledge (SK), Factual Knowledge (FK), and Beliefs (Bf) regarding cognitive health. SK had 3 questions, scored on a 4-point scale (maximum score: 12). FK included seven questions, each scored 0.5, 0.75, or 1, with a total possible score of 63. The belief domain consisted of five questions, rated on four or five points, with a potential total score of 68. Additionally, ten young adults outside the study completed the questionnaire twice to assess test-retest reliability, yielding a satisfactory interclass coefficient of 0.87, indicating good reliability.

***Procedure***

Data collection took place in university auditorium halls, with participants from the EG and CG seated separately in different auditoriums. Investigators provided both groups with a brief introduction to the session's purpose and schedule, ensuring a distraction-free environment. The EG watched an educational video on cognitive health, while the CG watched a pre-existing animated video of equal duration which was not related to brain health. Both videos were shown on a large screen with audio through loudspeakers. Participants from both groups completed a retrospective pre-post survey on cognitive health immediately after watching the videos. The flow diagram of the procedure is depicted in Figure 1.

Figure 1.

*Flow diagram of the study procedure*


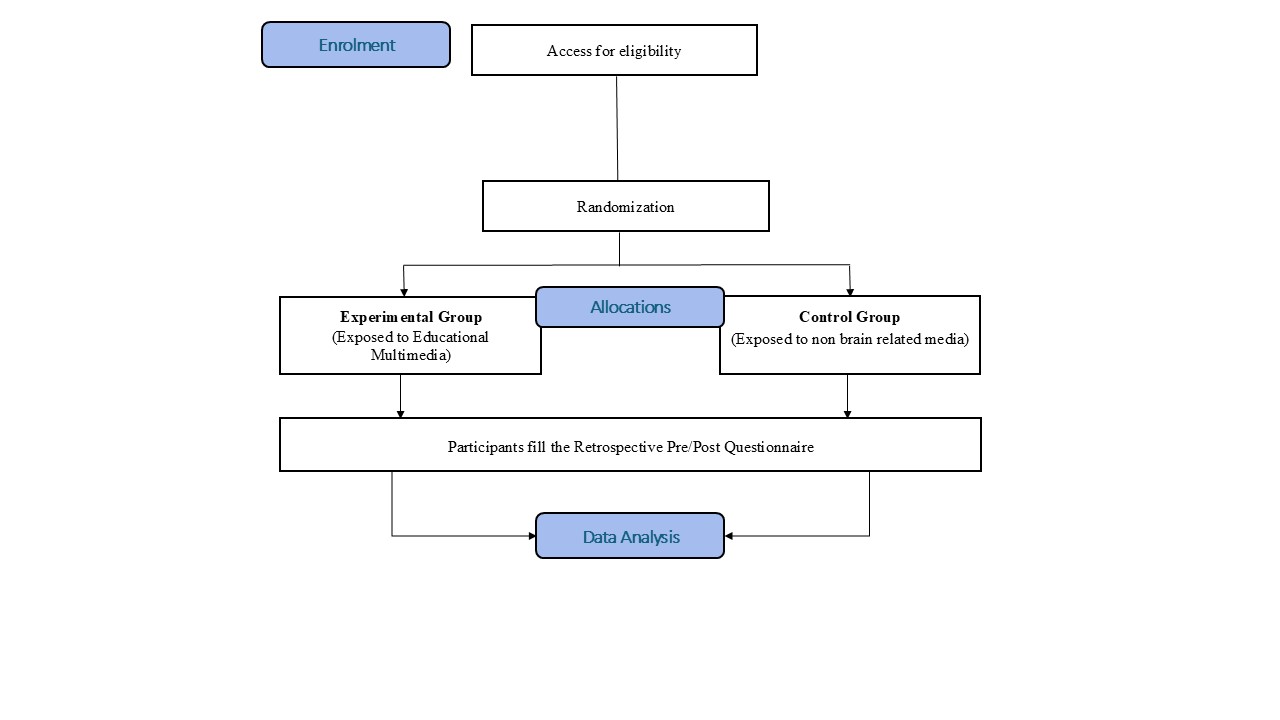


**Safety considerations**

This study involves minimal risk to participants, as it consists solely of viewing an educational video followed by completion of a questionnaire. To ensure psychological safety, the video content has been carefully designed to be informative without including any material likely to cause distress. All responses to the questionnaire will be kept confidential, and no personally identifiable information will be collected or linked to individual responses. Data will be securely stored to protect participant privacy. Participation in the study is voluntary, and all participants will provide informed consent prior to beginning. They will also be informed of their right to withdraw from the study at any time without any consequences. Given these precautions, we believe the study poses no physical or psychological harm to participants.

**Follow-up**

The present study did not have any follow-up sessions

**Data management and statistical analysis**

Data from the retrospective pre-post questionnaire were anonymized and coded in an Excel Sheet prior to analysis. Statistical analysis was done using Jamovi software (v2.3.21). Descriptive statistics were used to calculate the mean and standard deviation of the pre- and post-test scores on the SK, FK, and Bf domains. A mixed model ANOVA with a post hoc Bonferroni pairwise analysis with significance level set at p<0.05 was conducted to examine the effects of an EM intervention on knowledge and beliefs related to cognitive health between the EG and CG. Missing data were excluded from analysis to maintain the integrity of results and avoid potential biases. Excluding incomplete responses ensures that the findings accurately reflect only fully informed data points, providing a clearer assessment of the impact of the educational multimedia.

**Quality assurance**

Quality assurance for this study was maintained through adherence to established protocols and guidelines at all stages of the research process. Researchers and data collectors underwent training to ensure consistency in administering the questionnaire. Ongoing monitoring to identify any deviations from the protocol was carried out. For data verification processes, double data entry and routine audits was done to ensure the accuracy of data handling.

**Expected outcomes of the study**

The anticipated outcomes of this study include significant advancements in understanding how educational multimedia can enhance cognitive health literacy, as measured by improvements in Self-perceived Knowledge (SK), Factual Knowledge (FK), and Beliefs (Bf) scores. By providing evidence of the effectiveness of such interventions, the study aims to inform best practices in health education and communication strategies. The results will be disseminated through academic publications and presentations at conferences, ensuring that findings reach the research community. Moreover, we expect the outcomes to influence healthcare practices by equipping healthcare professionals with effective tools to educate patients about cognitive health. Additionally, the insights gained may inform health policy development, guiding the creation of initiatives that promote health literacy as a vital component of public health strategy, ultimately leading to improved health outcomes for individuals and communities

**Dissemination of results and publication policy**

Results from this study will be disseminated to a diverse audience, including healthcare professionals, researchers, and policymakers, to enhance awareness of the educational video’s effectiveness. We plan to publish the findings in scopus-indexed Q1 peer-reviewed journals and present them at relevant conferences to reach a wider academic audience. In addition, summary reports and community presentations will be developed to share results with participants and local stakeholders, ensuring that the insights gained are accessible and beneficial to those directly affected. We will also engage with policymakers to communicate the implications of our findings for health policy and practice, potentially influencing future initiatives aimed at enhancing cognitive health literacy. Regarding publication policy, Aysha Rooha will take the lead in manuscript, followed by Shreya Shetty, Aarushi Soni, Nidhi Lalu Jacob, Gagan Bajaj, Vinitha Mary George, and Jayashree S Bhat.

**Duration of the project**

The study spans from January 2022 and February 2023. The first three months (January-March) focused on finalizing the protocol. April-September focused on developing the educational multimedia and the retrospective pre-post questionnaire to assess knowledge and beliefs about cognitive health. October and November focused on recruitment of participants and data collection. December and January focused on data management, including data entry, data verification processes, and data analysis. The last month focused on preparation of manuscript.

**Problems anticipated**

Problems anticipated during the study include challenges in participant recruitment and retention. To mitigate these issues, we will implement a comprehensive outreach strategy and provide reminders to participants throughout the study. Data quality may also pose a challenge, as incomplete or inaccurate questionnaire responses could occur; we will enhance clarity in instructions and assure anonymity to encourage honest responses. Technical difficulties may arise during video presentation or data collection; thus, we will have backup equipment and alternative collection methods available.

**Project management**

All authors (AR, SS, AS, NLJ, GB, VMG, JSB) were involved in Conceptualization and designing the Methodology of the research idea. AR, SS and NLJ involved in the Investigation. Formal analysis by AR, SS, AS, GB. Project administration and Resources provided by GB, VMG and JSB. AR, SS, AS, and GB wrote the original draft. All authors (AR, SS, AS, NLJ, GB, VMG, JSB) contributed to refinement of the study protocol and approved the final manuscript.

**Ethics**

Ethical approval was obtained from the Institutional Ethics Committee (IEC KMC MLR 05-2022/171). Written informed consent was taken from all participants of the study prior to enrolment.

**REFERENCES**

1. Nichols, E. *et al.* Estimation of the global prevalence of dementia in 2019 and forecasted prevalence in 2050: an analysis for the Global Burden of Disease Study 2019. *Lancet Public Health* **7**, e105–e125 (2022).

2. World Health Organization. *Global Action Plan on the Public Health Response to Dementia 2017–2025*. (World Health Organization, Geneva, 2017).

3. Lanzi, A. M., Ellison, J. M. & Cohen, M. L. The “Counseling+” Roles of the Speech-Language Pathologist Serving Older Adults With Mild Cognitive Impairment and Dementia From Alzheimer’s Disease. *Perspect. ASHA Spec. Interest Groups* **6**, 987–1002 (2021).

4. Rudd, R. E. The evolving concept of Health literacy: New directions for health literacy studies. *J. Commun. Healthc.* **8**, 7–9 (2015).

5. Andrulis, D. P. & Brach, C. Integrating Literacy, Culture, and Language to Improve Health Care Quality for Diverse Populations. *Am. J. Health Behav.* **31**, S122–S133 (2007).

6. Abel, T., Hofmann, K., Ackermann, S., Bucher, S. & Sakarya, S. Health literacy among young adults: A short survey tool for public health and health promotion research. *Health Promot. Int.* **30**, (2014).

7. Sansom-Daly, U. M. *et al.* Health Literacy in Adolescents and Young Adults: An Updated Review. *J. Adolesc. Young Adult Oncol.* **5**, 106–118 (2016).

8. Greenlaw, C., Elhefnawy, Y., Jonas, R. & Douglass, L. M. Using an animated video to promote an informed discussion on SUDEP with adolescents. *Epilepsy Behav. EB* **122**, 108182 (2021).

9. Vandormael, A. *et al.* The Effect of a Wordless, Animated, Social Media Video Intervention on COVID-19 Prevention: Online Randomized Controlled Trial. *JMIR Public Health Surveill.* **7**, e29060 (2021).

10. Ani Petrosyan. Internet and social media users in the world 2023. *Statista* https://www.statista.com/statistics/617136/digital-population-worldwide/ (2023).

11. Simpson, A Rae. Young Adult Development Project. (2018).

12. Ustun, T. B., Kostanjesek, N., Chatterji, S., Rehm, J. & Organization, W. H. *Measuring Health and Disability : Manual for WHO Disability Assessment Schedule (WHODAS 2.0)*. https://apps.who.int/iris/handle/10665/43974 (2010).

13. Lincoln, K. D., Chow, T. W. & Gaines, B. F. BrainWorks: A Comparative Effectiveness Trial to Examine Alzheimer’s Disease Education for Community-Dwelling African Americans. *Am. J. Geriatr. Psychiatry Off. J. Am. Assoc. Geriatr. Psychiatry* **27**, 53–61 (2019).

14. Rooha, A. *et al.* Development and validation of educational multimedia to promote public health literacy about healthy cognitive aging. *Health Expect.* **n/a**, (2023).

15. Lu, Y., Liu, C., Fawkes, S., Wang, Z. & Yu, D. Knowledge, attitudes, and practice of general practitioners toward community detection and management of mild cognitive impairment: a cross-sectional study in Shanghai, China. *BMC Prim. Care* **23**, 114 (2022).

16. Bhanji, F., Gottesman, R., de Grave, W., Steinert, Y. & Winer, L. R. The retrospective pre-post: a practical method to evaluate learning from an educational program. *Acad. Emerg. Med. Off. J. Soc. Acad. Emerg. Med.* **19**, 189–194 (2012).

17. Ansari, Z. *et al.* Validity and Reliability of a Questionnaire Measuring Knowledge, Attitude, and Practice Regarding Dementia Among General Population and Healthcare Workers in Urban India. *Cureus* **14**, e28196 (2022).


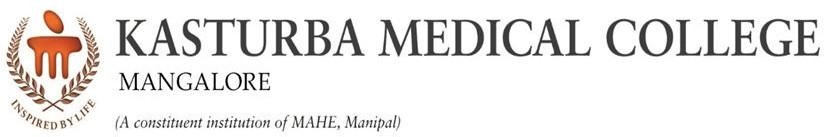


**PATIENT INFORMATION SHEET**

You are being invited to take part in a research study conducted by Ms. Shreya Shetty studying Masters in Speech-Language Pathology at Kasturba Medical College, Attavar, Mangalore. Before you decide whether to participate, it is important for you to understand why the research is being done and what it will involve. Please take time to read the following information carefully. You are being asked to participate in this research study because you are meeting the selection criteria.

**Why is this study being done?**

The purpose of the present study is to examine the efficacy of educational video on the beliefs of young-aged adults about cognitive health. The entire study may take up to 30-40 minutes for each person. All the tests administered are non-invasive.

**What will happen if I take part in this study?**

The researcher will collect information about you, which will be stored at Kasturba Medical College (KMC). If you agree to participate in this research, you are giving permission to use your health information for research. However, your information will only be used by KMC for research purposes only. Further, you may get or may not get the video to observe.

**Are there potential benefits to taking part in the study?**

The developed educational video will facilitate better understanding and importance of cognitive health.

**What are the risks of the study?**

There are no physical risks associated with this study. All the tests administered are noninvasive.

**What are the costs?**

There are no additional costs to you associated with participating in this research study.

**What about confidentiality?**

Information from this study will be reviewed only by authorized personnel or representatives, ethics committee or regulatory bodies who will be responsible for doing this research and drawing proper inferences and conclusions. Information and results from the study may be presented at meetings or published in journals without including your name and other personal identifications.

**What Are My Rights?**

Your participation in this research study is voluntary. You may choose not to be in the study. If you agree to be in the study, you may withdraw from the study at any time. If you withdraw from the study, no new data about you will be collected for research purposes.

Your decision not to participate or to withdraw from the study will not involve any penalty or loss of benefits. You will continue to receive your usual medical care from the hospital as well as the department whether or not you decide to participate in this study.

After you had a chance to read this information sheet and made a decision about whether you want to participate, please let the researcher know what you have decided. You are required to sign a consent form to participate in this research, but you must let the researcher know whether or not you wish to participate. If you have any questions regarding the research study or the information sheet, please ask the researcher. You will be given a copy of this information sheet to take home with you.

**Contact details:**

**Shreya Shetty: 9632074831**

**Dr. Gagan Bajaj:9886461351**


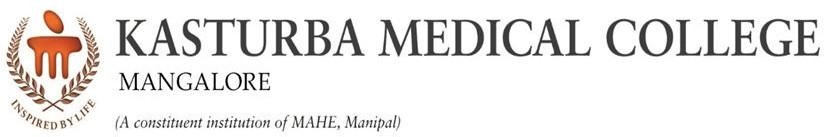


**INFORMED CONSENT FORM**

**Subject’s code:**

**Age / gender:**

I confirm that I have understood the information sheet for the study and have had the opportunity to ask questions. I understand that my participation in the study is voluntary and that I am free to withdraw at any time, without giving any reason, without my medical care or legal rights being affected. I understand that the Ethics Committee and the regulatory authorities will not need my permission to look at my health records both in respect of the current study and any further research that may be conducted in relation to it, even if I withdraw from the trial. However, I understand that my identity will not be revealed in any information released to third parties or published. I agree not to restrict the use of any data or results that arise from this study provided such use is only for the scientific purpose(s).

**
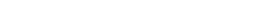

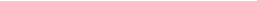
**

**Signature or thumb impression of the subject                       Signature of the Investigator**
